# Description of two cryptic species of the *Amolopsricketti* group (Anura, Ranidae) from southeastern China

**DOI:** 10.3897/zookeys.812.29956

**Published:** 2019-01-03

**Authors:** Zhi-Tong Lyu, Lin-Sheng Huang, Jian Wang, Yuan-Qiu Li, Hong-Hui Chen, Shuo Qi, Ying-Yong Wang

**Affiliations:** 1 State Key Laboratory of Biocontrol / The Museum of Biology, School of Life Sciences, Sun Yat-sen University, Guangzhou 510275, China Sun Yat-sen University Guangzhou China; 2 Guangdong Shimentai National Nature Reserve, Qingyuan 513000, China Guangdong Shimentai National Nature Reserve Qingyuan China; 3 Institute of herpetology, Shenyang Normal University, Shenyang 110034, China Shenyang Normal University Shenyang China

**Keywords:** *Amolopssinensis* sp. n., *Amolopsyatseni* sp. n., mitochondrial DNA, morphology, phylogeny, species complex, torrent frog

## Abstract

Two cryptic species, which were previously reported as *Amolopsricketti*, are revealed on the basis of significant morphological and genetic divergences. *Amolopssinensis***sp. n.** from central Guangdong, northeastern Guangxi and southwestern Hunan can be distinguished by the longitudinal glandular folds on the skin of the shoulders and other character combinations. *Amolopsyatseni***sp. n.** from the coastal hills of west Guangdong can be distinguished by the dense tiny round translucent, or white, spines on the dorsal skin of the body, dorsal and dorsolateral skin of the limbs, and other character combinations. The phylogenetic interrelationships of the *A.ricketti* group have been inferred as (*A.wuyiensis* + *A.ricketti*) + (*A.yunkaiensis* + (*A.albispinus* + (*A.sinensis***sp. n.** + *A.yatseni***sp. n.**))). This work indicates that the current records of *A.ricketti* might be a species complex composed of multiple species, and further work is needed to figure out this puzzle.

## Introduction

The torrent frog genus *Amolops* Cope, 1865, which is comprised of 55 species, is widespread from the southern and eastern Himalayas, eastward to southeastern mainland China, and southward to Malay Peninsula (Frost 2018). Of these, 28 species from China were assigned into six species groups (Fei et al. 2009, 2017; Sun et al. 2013; Lu et al. 2014; Jiang et al. 2016; Sung et al. 2016; Yuan et al. 2018; Lyu et al. 2018). Among them, the *A.ricketti* group is a monophyletic species group containing four recognized species from southeastern China (Fei et al. 2012; Sung et al. 2016; Lyu et al. 2018): *A.yunkaiensis* Lyu, Wang, Liu, Zeng & Wang, 2018 from southwestern Guangdong, *A.albispinus* Sung, Wang & Wang, 2016 from Shenzhen City, Guangdong, *A.wuyiensis* (Liu & Hu, 1975) distributed in Fujian, Zhejiang, Anhui and Jiangxi in eastern China, and *A.ricketti* (Boulenger, 1899) reported to be widely distributed in Sichuan, Chongqing, Yunnan, Guizhou, Hubei, Hunan, Jiangxi, Fujian, Guangdong and Guangxi in southern China and even to northern and central Indochina. It is worth noting that the species *A.albispinus* and *A.yunkaiensis* were recognized as other known species for a long time and recognition as new species represented the beginning of uncovering the cryptic diversity within the *A.ricketti* group (Sung et al. 2016; Lyu et al. 2018).

During our herpetological surveys in Guangdong, Guangxi and Hunan provinces in southeastern China, we have collected a series of *Amolops* specimens which were recorded as *A.ricketti* (Fei et al. 2009, 2012; Li et al. 2011). However, morphological examinations indicated that these specimens belong to two different undescribed species that can be markedly and reliably distinguished from all congeners, especially from specimens of *A.ricketti* collected from the type locality, Mt. Wuyi, Fujian. Molecular analyses further well supported the morphological result, showing that these specimens formed two lineages within the *A.ricketti* group; in this study we describe them as two new species of genus *Amolops*.

## Material and methods

### Sampling

**Figure 1.**
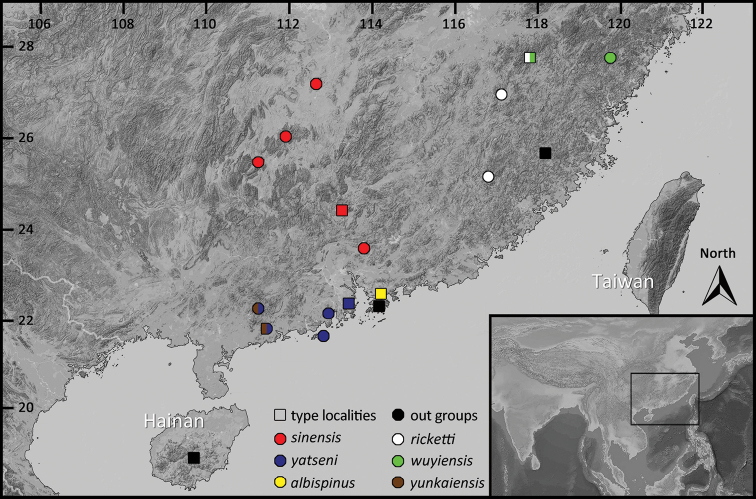
Collecting localities of samples used in this study.

A total of 28 muscle samples of the two new species were collected for molecular analyses, encompassing six from Zhongshan City, Guangdong, two from Shangchuan Island, Guangdong, two from Mt. Gudou, Guangdong, three from Ehuangzhang Nature Reserve, Guangdong, two from Yunkaishan Nature Reserve, Guangdong, seven from Shimentai Nature Reserve, Guangdong, two from Mt. Nankun, Guangdong, two from Mt. Dupangling, Guangxi, one from Mt. Yangming, Hunan, and one from Mt. Hengshan, Hunan. In addition, 36 samples from nine known species of the genus *Amolops*, namely *A.albispinus*, *A.ricketti*, *A.wuyiensis*, *A.yunkaiensis*, *A.daiyunensis* (Liu & Hu, 1975), *A.hongkongensis* (Pope & Romer, 1951), *A.hainanensis*(Boulenger, 1900), *A.torrentis* (Smith, 1923) and *A.chunganensis* (Pope, 1929), were also collected and incorporated into our dataset. All muscle samples were attained from euthanasia specimens and then preserved in 95% ethanol and stored at -40 °C. Detail information for these materials is shown in Table 1 and Figure 1.

**Table 1.** Localities, voucher number and GenBank numbers for all samples used in this study.

**ID**	**Species**	Localities (* type localities)	**Voucher**	**16S**	**CO1**
1	*Amolopssinensis* sp. n.	* China: Shimentai Nature Reserve, Guangdong	SYS a004165	MK263262	MK263314
2		* China: Shimentai Nature Reserve, Guangdong	SYS a004722	MK263278	MK263318
3		* China: Shimentai Nature Reserve, Guangdong	SYS a007105	MK263297	MK263329
4		* China: Shimentai Nature Reserve, Guangdong	SYS a007106	MK263298	MK263330
5		* China: Shimentai Nature Reserve, Guangdong	SYS a007107	MK263299	MK263331
6		* China: Shimentai Nature Reserve, Guangdong	SYS a007108	MK263300	MK263332
7		* China: Shimentai Nature Reserve, Guangdong	SYS a007109	MK263301	MK263333
8		China: Mt. Nankun, Guangdong	SYS a005710	MK263287	MK263321
9		China: Mt. Nankun, Guangdong	SYS a005712	MK263288	MK263322
10		China: Mt. Dupangling, Guangxi	SYS a005089	MK263279	MK263319
11		China: Mt. Dupangling, Guangxi	SYS a005111	MK263280	MK263320
12		China: Mt. Yangming, Hunan	SYS a007268	MK263302	MK263334
13		China: Mt. Hengshan, Hunan	SYS a004257	MK263265	MK263315
14	*Amolopsyatseni* sp. n.	* China: Zhongshan City, Guangdong	SYS a006806	MK263289	MK263323
15		* China: Zhongshan City, Guangdong	SYS a006807	MK263290	MK263324
16		* China: Zhongshan City, Guangdong	SYS a006808	MK263291	MK263325
17		* China: Zhongshan City, Guangdong	SYS a006810	MK263292	MK263326
18		* China: Zhongshan City, Guangdong	SYS a006811	MK263293	MK263327
19		* China: Zhongshan City, Guangdong	SYS a006857	MK263296	MK263328
20		China: Shangchuan Island, Guangdong	SYS a003633	MK263250	MK263304
21		China: Shangchuan Island, Guangdong	SYS a003634	MK263251	MK263305
22		China: Mt. Gudou, Guangdong	SYS a006818	MK263294	MK263306
23		China: Mt. Gudou, Guangdong	SYS a006819	MK263295	MK263307
24		China: Ehuangzhang Nature Reserve, Guangdong	SYS a003978	MK263252	MK263308
25		China: Ehuangzhang Nature Reserve, Guangdong	SYS a003980	MK263254	MK263309
26		China: Ehuangzhang Nature Reserve, Guangdong	SYS a003981	MK263255	MK263310
27		China: Yunkaishan Nature Reserve, Guangdong	SYS a004642	MK263269	MK263316
28		China: Yunkaishan Nature Reserve, Guangdong	SYS a004643	MK263270	MK263317
29	* Amolops albispinus *	* China: Mt. Wutong, Guangdong	SYS a003452	MK263247	KX507332
30		* China: Mt. Wutong, Guangdong	SYS a003453	MK263248	KX507333
31		* China: Mt. Wutong, Guangdong	SYS a003454	MK263249	KX507334
32	* Amolops ricketti *	* China: Mt. Wuyi, Fujian	SYS a004141	MK263259	MG991927
33		* China: Mt. Wuyi, Fujian	SYS a004142	MK263260	MG991928
34		* China: Mt. Wuyi, Fujian	SYS a004143	MK263261	MG991929
35		China: Mt. Emeifeng, Fujian	SYS a002492	MK263244	KX507329
36		China: Shanghang County, Fujian	SYS a003342	MK263246	KX507331
37		China: Shanghang County, Fujian	SYS a004106	MK263256	MK263311
38	* Amolops wuyiensis *	* China: Mt. Wuyi, Fujian	SYS a001716	MK263239	KX507324
39		* China: Mt. Wuyi, Fujian	SYS a001717	MK263240	KX507325
40		* China: Mt. Wuyi, Fujian	SYS a004139	MK263257	MK263312
41		* China: Mt. Wuyi, Fujian	SYS a004140	MK263258	MK263313
42		China: Jingning County, Zhejiang	SYS a002723	MK263245	MK263303
43	* Amolops yunkaiensis *	* China: Ehuangzhang Nature Reserve, Guangdong	SYS a003979	MK263253	MG991907
44		* China: Ehuangzhang Nature Reserve, Guangdong	SYS a004705	MK263275	MG991906
45		* China: Ehuangzhang Nature Reserve, Guangdong	SYS a004706	MK263276	MG991908
46		* China: Ehuangzhang Nature Reserve, Guangdong	SYS a004707	MK263277	MG991909
47		China: Yunkaishan Nature Reserve, Guangdong	SYS a004681	MK263271	MG991910
48	* Amolops yunkaiensis *	China: Yunkaishan Nature Reserve, Guangdong	SYS a004682	MK263272	MG991911
49		China: Yunkaishan Nature Reserve, Guangdong	SYS a004683	MK263273	MG991912
50		China: Yunkaishan Nature Reserve, Guangdong	SYS a004684	MK263274	MG991913
51	* Amolops daiyunensis *	* China: Mt. Daiyun, Fujian	SYS a001737	MK263241	KX507326
52		* China: Mt. Daiyun, Fujian	SYS a001738	MK263242	KX507327
53		* China: Mt. Daiyun, Fujian	SYS a001739	MK263243	KX507328
54	* Amolops hongkongensis *	* China: Hong Kong	SYS a004577	MK263266	MG991919
55		* China: Hong Kong	SYS a004578	MK263267	MG991920
56		* China: Hong Kong	SYS a004579	MK263268	MG991921
57	* Amolops hainanensis *	* China: Mt. Wuzhi, Hainan	SYS a005281	MK263281	MG991916
58		* China: Mt. Wuzhi, Hainan	SYS a005282	MK263282	MG991917
59		* China: Mt. Wuzhi, Hainan	SYS a005283	MK263283	MG991918
60	* Amolops torrentis *	* China: Mt. Wuzhi, Hainan	SYS a005289	MK263284	MG991930
61		* China: Mt. Wuzhi, Hainan	SYS a005290	MK263285	MG991931
62		* China: Mt. Wuzhi, Hainan	SYS a005291	MK263286	MG991932
63	* Amolops chunganensis *	China: Mt. Jinggang, Jiangxi	SYS a004212	MK263263	MG991914
64		China: Mt. Jinggang, Jiangxi	SYS a004213	MK263264	MG991915

### DNA Extraction, PCR and sequencing

Genomic DNA was extracted from muscular tissue by using a DNA extraction kit from Tiangen Biotech (Beijing) Co., Ltd. Partial 16S ribosomal RNA gene (16S) and partial cytochrome C oxidase 1 gene (CO1) were amplified. Primers used for 16S were L3975 (5’-CGCCTGTTTACCAAAAACAT-3’) and H4551 (5’-CCGGTCTGAACTCAGATCACGT-3’) following Simon et al. (1994), and L2A (5’-CCAAACGAGCCTAGTGATAGCTGGTT-3’) and H10 (5’-TGATTACGCTACCTTTGCACGGT-3’) following Chen et al. (2013), and for CO1 were Chmf4 (5’-TYTCWACWAAYCAYAAAGAYATCGG-3’) and Chmr4 (5’-ACYTCRGGRTGRCCRAARAATCA-3’) following Che et al. (2012), and dgLCO (5’-GGTCAACAAATCATAAAGAYATYGG-3’) and dgHCO (5’-AAACTTCAGGGTGACCAAARAAYCA-3’) following Meyer et al. (2005). PCR amplifications were processed in a 20 reaction volume with the cycling conditions as follows: an initial denaturing step at 95 °C for 4 min, 35 cycles of denaturing at 94 °C for 40 s, annealing at 53 °C (for 16S) / 48 °C (for CO1) for 40 s and extending at 72 °C for 1 min, and final extending step of 72 °C for 10 min. PCR products were purified with spin columns. The purified products were sequenced with the same primers using a BigDye Terminator Cycle Sequencing Kit as per the guidelines, on an ABI Prism 3730 automated DNA sequencer by Shanghai Majorbio Bio-pharm Technology Co., Ltd and Beijing Genomics Institute. All sequences were deposited in GenBank (Table 1).

### Phylogenetic analyses

DNA sequences were aligned by the Clustal W algorithm with default parameters (Thompson et al. 1997) and trimmed with the gaps partially deleted in MEGA 6 (Tamura et al. 2013), while within highly variable regions, all gaps were removed. Two gene segments, 637 base pairs (bp) of CO1 and 1032 bp of 16S, were concatenated seriatim into a 1669-bp sequence, and further divided into two partitions based upon each gene. Two partitions were tested respectively in jmodeltest v2.1.2 (Darriba et al. 2012) with Akaike and Bayesian information criteria, all resulting in the best-fitting nucleotide substitution models of GTR + I + G. Sequenced data were analyzed using Bayesian inference (BI) in MrBayes 3.2.4 (Ronquist et al. 2012) and maximum likelihood (ML) in RaxmlGUI 1.3 (Silvestro and Michalak 2012). Two independent runs were conducted in a BI analysis, each of which was performed for 2,000,000 generations and sampled every 1000 generations with the first 25% samples discarded as burn-in, resulting a potential scale reduction factor (PSRF) of < 0.01. In ML analysis, the bootstrap consensus tree inferred from 1000 replicates was used to represent the evolutionary history of the taxa analyzed. Pairwise distances (*p*-distance) were calculated in MEGA 6 using the uncorrected p-distance model.

### Morphology

We obtained diagnostic characters of known species of the genus *Amolops* from the literature for comparison. In addition, a total of 67 museum specimens of *A.ricketti* group were examined for comparison, which are listed in Appendix 1.

Measurements follow Fei et al. (2009) and Lyu et al. (2018), and were taken with digital calipers (Neiko 01407A Stainless Steel 6-Inch Digital Caliper, USA) to the nearest 0.1 mm. These measurements are as follows:

**SVL** snout-vent length (from tip of snout to posterior margin of vent);

**HDL** head length (from tip of snout to the articulation of the jaw);

**HDW** head width (head width at the commissure of the jaws);

**SNT** snout length (from tip of snout to the anterior corner of the eye);

**IND** internasal distance (distance between nares);

**IOD** interorbital distance (minimum distance between upper eyelids);

**ED** eye diameter (from the anterior corner of the eye to posterior corner of the eye);

**TD** tympanum diameter (horizontal diameter of tympanum);

**TED** tympanum-eye distance (from anterior edge of tympanum to posterior corner of the eye);

**HND** hand length (from distal end of radioulna to tip of distal phalanx III);

**RAD** radioulna length (from the flexed elbow to the base of the outer palmar tubercle);

**FTL** foot length (from distal end of tibia to tip of distal phalanx IV);

**TIB** tibial length (from the outer surface of the flexed knee to the heel);

**F3W** width of digital disc on finger III;

**T4W** width of digital disc on toe IV.

Sex was determined by observation of secondary sexual characters, i.e. the presence of nuptial spines in males, following Fei et al. (2009) and Lyu et al. (2018).

All specimens were fixed in 10% buffered formalin and later transferred to 70% ethanol, and deposited in The Museum of Biology, Sun Yat-sen University (SYS) and Chengdu Institute of Biology, the Chinese Academy of Sciences (CIB), P.R. China.

## Results

**Figure 2.**
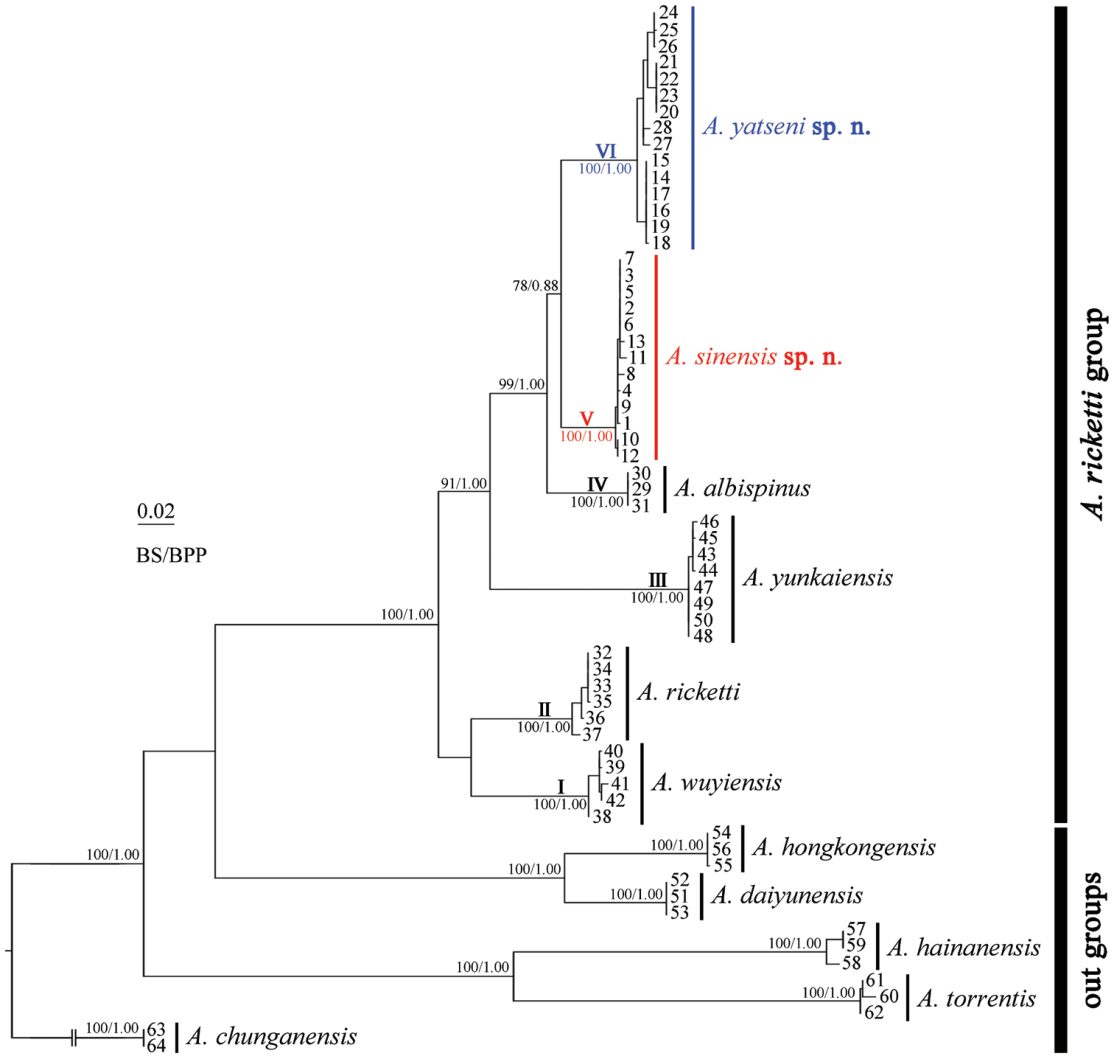
Bayesian inference and maximum-likelihood phylogenies. The bootstrap supports for maximum likelihood analysis (BS) > 75 and the Bayesian posterior probabilities (BPP) > 0.85 were retained.

The phylogenetic trees strongly supported that the *Amolopsricketti* group was a monophyletic species group, which can be further divided into six well-supported clades with marked divergences (Fig. 2; Table 2). The four known species of this group, *A.wuyiensis*, *A.ricketti*, *A.yunkaiensis* and *A.albispinus*, formed the four basal clades respectively. The unnamed specimens from Shimentai Nature Reserve and Mt. Nankun in Guangdong, Mt. Dupangling in Guangxi, and Mt. Yangming and Mt. Hengshan in Hunan clustered into a clade (clade V) with highly supported node values (BS = 100, BPP = 1.00) and small divergences (*p*-distance 0.0–0.6); the specimens from Zhongshan City, Mt. Gudou, Shangchuan Island, Ehuangzhang Nature Reserve and Yunkaishan Nature Reserve clustered into a clade (clade VI) with highly supported node values (BS = 100, BPP = 1.00) and small divergences (*p*-distance 0.0–0.8). These two clades are sister taxa to each other with significant divergences (*p*-distance 3.5–4.2).

**Table 2.** Uncorrected *p*-distances (in %) among the *Amolops* species in this study.

ID	Species	I	II	III	IV	V	VI	VII	IIX	IX	X	XI
**I**	*Amolopssinensis* sp. n.	0.0–0.6	–	–	–	–	–	–	–	–	–	–
**II**	*Amolopsyatseni* sp. n.	3.5–4.2	0.0–0.8	–	–	–	–	–	–	–	–	–
**III**	* Amolops albispinus *	3.8–4.1	4.1–4.4	0.0–0.0	–	–	–	–	–	–	–	–
**IV**	* Amolops ricketti *	6.7–7.2	6.4–7.0	6.4–6.7	0.0–0.7	–	–	–	–	–	–	–
**V**	* Amolops wuyiensis *	6.2–6.8	6.2–6.8	6.8–7.0	5.1–5.5	0.1–0.5	–	–	–	–	–	–
**VI**	* Amolops yunkaiensis *	6.4–6.9	6.9–7.7	6.7–7.0	7.9–8.2	8.0–8.3	0.0–0.2	–	–	–	–	–
**VII**	* Amolops daiyunensis *	12.5–12.9	12.6–12.7	12.4–12.4	12.3–12.8	12.6–12.8	12.4–12.6	0.0–0.0	–	–	–	–
**IIX**	* Amolops hongkongensis *	12.5–12.9	12.2–12.4	12.2–12.3	11.8–12.3	12.1–12.4	12.4–12.7	5.5–5.5	0.0–0.1	–	–	–
**IX**	* Amolops hainanensis *	15.1–15.5	15.4–15.8	14.6–14.7	14.9–15.2	14.5–14.8	15.6–16.0	14.8–14.9	15.7–15.8	0.0–0.8	–	–
**X**	* Amolops torrentis *	15.3–15.7	15.5–15.8	15.5–15.5	15.1–15.5	15.3–15.5	16.3–16.7	14.8–15.0	14.6–14.7	10.9–11.0	0.1–0.4	–
**XI**	* Amolops chunganensis *	15.8–16.2	15.6–16.0	15.8–15.8	14.8–15.2	14.8–15.0	15.3–15.5	15.3–15.3	15.8–15.9	17.7–17.9	17.3–17.5	0.0

Morphologically, the specimens clustered into clade V from central Guangdong, northeastern Guangxi and southwestern Hunan can be distinguished from all known *Amolops* species by having unique longitudinal glandular folds on the skin of the shoulders and other characters (see diagnosis below). Therefore this clade represents a distinct evolutionary lineage, and is described below as a new species, *Amolopssinensis* sp. n. The specimens grouped in clade VI from the coastal hills of west Guangdong differ from all known *Amolops* species by having unique dense tiny round translucent, or white, spines on the dorsal skin of the body, dorsal and dorsolateral skin of the limbs and other characters (see diagnosis below). Therefore, this clade represents a distinct evolutionary lineage, and is described below as a new species *Amolopsyatseni* sp. n.

### Taxonomy accounts

#### Amolops sinensis

Taxon classificationAnimaliaAnuraRanidae

Lyu, Wang & Wang sp. n.

http://zoobank.org/DF35246E-39C3-46E7-8C16-F2ACD366C01C

##### Chresonymy.

*Amolopsricketti* (Boulenger, 1899): Fei et al. 2009 (Hengshan, Hunan); Li et al. 2011 (Guangdong); Fei et al. 2012 (Guangxi; Hengshan, Hunan).

##### Holotype.

**Figure 3.**
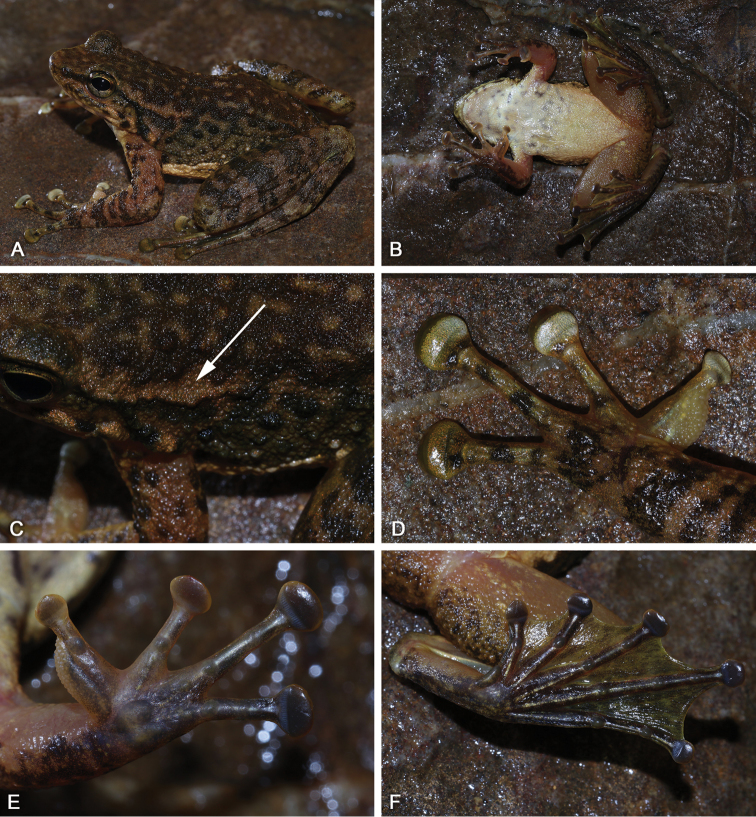
Morphological features of the adult male holotype SYS a007107 of *Amolopssinensis* sp. n. in life. **A** Dorsolateral view **B** Ventral view **C** A longitudinal glandular folds on skin of shoulder **D** Beige nuptial pad and nuptial spines **E** Left hand **F** Left foot.

SYS a007107 (Fig. 3), adult male, collected by Hong-Hui Chen (HHC) and Yuan-Qiu Li (YQL) on 22 June 2018 from Qianjin (24.49N, 113.11E; ca 510 m a.s.l.), Shimentai Nature Reserve, Guangdong.

##### Paratypes.

Ten adult specimens. Male SYS a007105, 7106 and 7108, and female SYS a007109, collected at the same time from the same stream as holotype. Female SYS a004165/CIB 110004, collected by Zhi-Tong Lyu (ZTL) and YQL on 26 July 2015 from Hengshitang, Shimentai Nature Reserve, Guangdong. Male SYS a005710 and female SYS a005712, collected by ZTL and Jian Wang (JW) on 8 April 2017 from Mt. Nankun, Guangdong. Male SYS a005089, collected by JW on 18 July 2016 from Mt. Dupangling, Guangxi. Female SYS a007268, collected by ZTL and Yu-Long Li on 21 June 2018 from Mt. Yangming, Hunan. Female SYS a004257, collected by ZTL and JW on 19 August 2015 from Mt. Hengshan, Hunan.

##### Other examined material.

**Figure 4.**
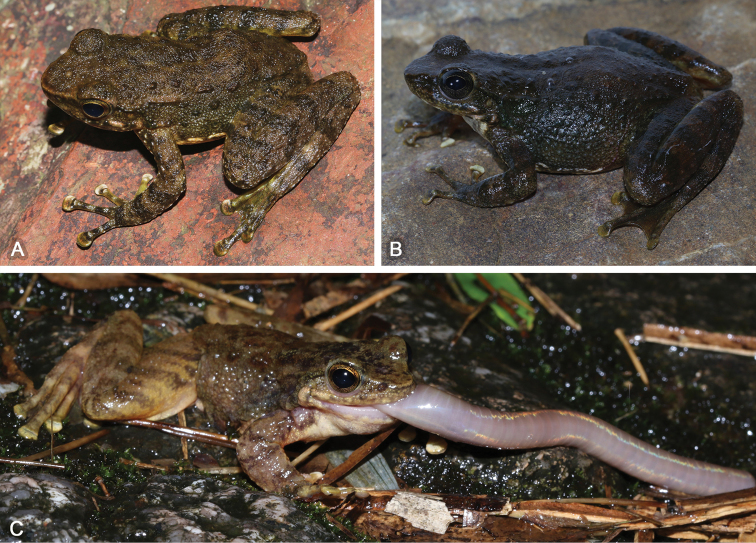
**A** Juvenile SYS a004722 of *Amolopssinensis* sp. n. in life **B** Female paratype SYS a007109 in life **C** Male paratype SYS a005710 in life, eating an earthworm.

Juvenile SYS a004722 (Fig. 4A), collected by ZTL, JW and YQL on 1 May 2016 from the same stream as holotype.

##### Diagnosis.

The new species was assigned to genus *Amolops* and further to the *A.ricketti* group morphologically based on the absence of dorsolateral folds, the presence of a circummarginal groove on the disk of the first finger, the absence of tarsal glands, and the presence of nuptial pads with conical nuptial spines on the first finger in males.

*Amolopssinensis* sp. n. is distinguished from its congeners by a combination of the following morphological characteristics: (1) body stout and robust, SVL 40.2–46.5 (43.1±2.2, n=6) mm in adult males, 47.7–52.7 (50.5±2.0, n=5) mm in adult females; (2) dorsal body olive-brown to dark brown, with irregular light strip-shaped patches or not; (3) ventral surface creamy white or beige, with dark gray patches; (4) dorsal skin of body very rough, granular and scattered with conical tubercles and raised large warts in males; (5) vomerine teeth strong, tongue cordiform, deeply notched posteriorly; (6) absence of dorsolateral folds; (7) a longitudinal glandular fold on skin of shoulder on each side; (8) supernumerary tubercles below base of fingers III and IV indistinct; (9) heels overlapping; (10) absence of outer metatarsal tubercles and tarsal glands; (11) absence of vocal sacs; (12) nuptial pad on first finger prominent with beige spines in breeding males; and (13) white conical spines present on skin of temporal region (including tympanum in several individuals) and loreal region in breeding males.

##### Description of holotype.

Body stout, SVL 43.3 mm. Head width slightly smaller than head length (HDW/HDL = 1.04); snout short (SNT/HDL = 0.45) and rounded in profile, projecting beyond lower jaw; nostril closer to tip of snout than eye; loreal region concave; top of head flat; eye large (ED/HDL = 0.32) and convex; canthus rostralis distinct; pineal body distinct; tympanum small, edge distinct; tympanum-eye distance smaller than tympanum, TED/TD 0.90; supratympanic fold distinct, start from back of eye and extending to shoulder, a well-developed gland on end of supratympanic fold; choanae moderate; vomerine teeth present; tongue cordiform, deeply notched posteriorly.

Forelimbs moderately robust; hands moderately long (HND/SVL = 0.33); relative finger lengths I = II < IV < III; finger tips dilated to wide oval disks with circummarginal grooves, relative width of finger disks I < II < III = IV; subarticular tubercles prominent, rounded; supernumerary tubercles below base of fingers III and IV indistinct, below base of fingers I and II absent; inner metacarpal tubercle small, outer metacarpal tubercle prominent and slightly separated; absence of webbing and presence of weak lateral fringes on fingers.

Hindlimbs long and robust (TIB/SVL = 0.60); tibio-tarsal articulation reaching tip of snout when hindlimb stretched alongside of body; relative toe lengths I < II < III = V < IV; tips of all toes expanded to well-developed oval discs with circummarginal grooves; subarticular tubercles oval and distinct; inner metatarsal tubercle prominent, elongated; outer metatarsal tubercles absent; toes fully webbed; lateral fringes of toes I and V developed; tarsal glands absent; heels overlapping when hindlimbs flexed at right angles to axis of body.

Dorsal skin of body rough, granular and scattered with raised tubercles and warts; underdeveloped conical spines on skin of loreal region and temporal region except tympanum; flanks very rough and granular with raised warts; dorsal limbs rough with numerous tubercles; several longitudinal dermal ridges on dorsal surfaces of thigh, tibia and tarsus; dorsolateral fold absent; a longitudinal glandular fold on skin of shoulder; posterior part of upper lip swollen; rictal gland prominent and ellipsoidal, posterior to corner of mouth.

Ventral surface slightly wrinkled with granules; ventral surface of hand smooth, ventral surface of foot granular; large warts surrounding cloaca.

##### Measurements of holotype

**(in mm).**SVL 43.3; HDL 15.4, HDW 16.0; SNT 6.9; IND 6.2; IOD 4.3; ED 4.9; TD 1.9; TED 1.7; HND 14.3; RAD 9.4; FTL 36.9; TIB 26.0; F3W 2.9; T4W 2.9.

##### Color of holotype in life.

Dorsal body olive-brown with irregular light yellow patches; longitudinal glands on occipital region light yellow; warts on flanks dark or grayish white; irregular dark patches on dorsal surface of forearms, distinct dark transverse bars on dorsal surface of lower arms and hindlimbs; dorsal discs of digits brown or white; nuptial pads and nuptial spines beige; posterior edge of upper lip and rictal gland light maize-yellow; throat and chest creamy white; belly beige; several dark gray mottling on surface of throat, chest and anterior part of abdomen; ventral surfaces of limbs gray pink grounding; ventral hands and feet dark grey; warts around cloaca yellowish white tubercles and olive-brown.

##### Color of holotype in preservative.

Dorsal surface dark brown with irregular gray patches, transverse bars on limbs more distinct; longitudinal glands on occipital region more distinct; webs on toes gray, mottling with olive-brown; ventral surface grayish white, mottling on surface of throat, chest and anterior part of abdomen become more distinct; ventral surface of limbs beige.

##### Variations.

Measurements of type series specimens are given in Table 3. All specimens are very similar in morphology except that: dorsal skin dark brown without any patterns in SYS a007109 (Fig. 4B); skin of tympanum with white conical spines in SYS a005710 (Fig. 4C); nuptial spines are conical in SYS a005710; tibia-tarsal articulation reaching forward to the loreal region in SYS a004257, 5712, 7109 and 7268.

**Table 3.** Measurements (in mm; minimum-maximum, mean±1SD) of the type series of *Amolopssinensis* sp. n. and *A.yatseni* sp. n.

	*A.sinensis* sp. n.		*A.yatseni* sp. n.	
	Males (n=6)	Females (n=5)	Males (n=6)	Females (n=11)
SVL	40.2–46.5 (43.1±2.2)	47.7–52.7 (50.5±2.0)	39.3–44.7 (42.5±2.1)	42.1–48.9 (46.4±2.0)
HDL	14.6–16.7 (15.5±0.7)	16.1–18.6 (17.1±1.2)	14.2–16.8 (16.0±1.0)	15.4–18.1 (16.7±0.9)
HDW	15.2–18.4 (16.1±1.2)	16.2–19.5 (17.7±1.6)	16.1–17.8 (16.9±0.7)	16.1–19.0 (17.5±0.9)
SNT	6.0–7.5 (6.6±0.6)	6.6–7.9 (7.1±0.5)	5.6–7.4 (6.4±0.6)	6.2–7.2 (6.7±0.4)
IND	5.3–6.9 (5.9±0.5)	5.6–7.4 (6.6±0.8)	5.7–6.4 (6.1±0.3)	5.7–6.6 (6.3±0.3)
IOD	4.2–4.7 (4.4±0.2)	4.5–5.4 (4.8±0.4)	3.9–4.8 (4.3±0.3)	4.1–4.5 (4.3±0.1)
ED	4.8–5.6 (5.1±0.4)	5.4–6.4 (6.0±0.4)	4.7–5.3 (5.1±0.3)	4.9–6.2 (5.5±0.4)
TD	1.8–2.2 (2.1±0.2)	1.8–2.2 (2.1±0.1)	1.5–1.9 (1.7±0.1)	1.8–2.3 (2.0±0.2)
TED	1.4–1.8 (1.7±0.1)	1.5–2.0 (1.7±0.2)	1.4–2.0 (1.8±0.2)	1.8–2.9 (2.1±0.3)
HND	12.0–14.3 (13.0±0.8)	12.5–14.7 (13.7±0.9)	11.6–13.8 (12.7±0.9)	11.8–14.0 (12.9±0.6)
RAD	8.4–10.6 (9.3±0.7)	9.3–10.9 (10.0±0.7)	7.8–10.9 (8.9±1.1)	8.2–9.9 (8.8±0.5)
FTL	30.7–37.1 (34.3±2.4)	34.8–39.5 (36.9±1.9)	27.1–37.0 (32.4±3.3)	29.1–35.6 (33.2±2.4)
TIB	22.4–26.9 (24.7±1.7)	24.6–29.2 (26.9±1.8)	20.6–27.0 (24.5±2.5)	21.5–26.3 (24.6±1.5)
F3W	2.4–2.9 (2.7±0.2)	2.5–3.2 (2.9±0.3)	2.6–3.4 (2.8±0.3)	2.2–3.1 (2.8±0.3)
T4W	2.3–2.9 (2.5±0.2)	2.5–3.1 (2.8±0.3)	2.1–3.2 (2.4±0.4)	2.0–2.8 (2.4±0.3)
HDL/SVL	0.35–0.37 (0.36±0.01)	0.33–0.36 (0.34±0.01)	0.36–0.38 (0.38±0.01)	0.35–0.37 (0.36±0.01)
HDW/SVL	0.35–0.40 (0.37±0.02)	0.33–0.38 (0.35±0.02)	0.39–0.42 (0.40±0.01)	0.36–0.39 (0.38±0.01)
HDW/HDL	1.00–1.11 (1.04±0.04)	1.01–1.05 (1.03±0.02)	1.02–1.13 (1.06±0.04)	1.02–1.09 (1.05±0.02)
SNT/HDL	0.39–0.45 (0.43±0.02)	0.40–0.43 (0.42±0.01)	0.38–0.44 (0.40±0.02)	0.36–0.44 (0.40±0.02)
SNT/SVL	0.14–0.16 (0.15±0.01)	0.13–0.15 (0.14±0.01)	0.14–0.17 (0.15±0.01)	0.13–0.15 (0.15±0.01)
IND/HDW	0.34–0.39 (0.37±0.02)	0.35–0.39 (0.38±0.02)	0.33–0.38 (0.36±0.02)	0.34–0.39 (0.36±0.01)
IOD/HDW	0.26–0.30 (0.28±0.02)	0.26–0.28 (0.27±0.01)	0.23–0.28 (0.25±0.02)	0.24–0.26 (0.25±0.01)
ED/HDL	0.31–0.37 (0.33±0.02)	0.33–0.38 (0.35±0.02)	0.31–0.33 (0.32±0.01)	0.30–0.36 (0.33±0.02)
ED/SVL	0.11–0.13 (0.12±0.01)	0.11–0.12 (0.12±0.00)	0.12–0.12 (0.12±0.00)	0.11–0.13 (0.12±0.01)
TD/ED	0.36–0.46 (0.40±0.04)	0.29–0.39 (0.35±0.04)	0.28–0.37 (0.34±0.03)	0.32–0.40 (0.36±0.03)
TED/TD	0.78–0.90 (0.82±0.04)	0.74–0.97 (0.83±0.08)	0.81–1.18 (1.05±0.13)	0.86–1.61 (1.08±0.21)
HND/SVL	0.28–0.33 (0.30±0.02)	0.24–0.28 (0.27±0.02)	0.28–0.32 (0.30±0.01)	0.26–0.29 (0.28±0.01)
RAD/SVL	0.21–0.23 (0.22±0.01)	0.19–0.21 (0.20±0.01)	0.18–0.24 (0.21±0.02)	0.18–0.21 (0.19±0.01)
FTL/SVL	0.76–0.85 (0.80±0.03)	0.71–0.75 (0.73±0.02)	0.69–0.83 (0.76±0.04)	0.66–0.77 (0.72±0.03)
TIB/SVL	0.56–0.60 (0.57±0.01)	0.50–0.55 (0.53±0.02)	0.52–0.61 (0.57±0.03)	0.48–0.56 (0.53±0.02)

##### Sexual dimorphism.

*Amolopssinensis* sp. n. possesses distinct sexual dimorphism: (1) larger body size in females with SVL 47.7–52.7 mm (vs. SVL 40.2–46.5 mm in males); (2) beige nuptial spines on beige nuptial pads in breeding males; (3) dense white conical spines present on skin of temporal region and loreal region in males during breeding season (vs. absent in females); and (4) females bearing light yellow oocytes.

##### Comparisons.

**Figure 5.**
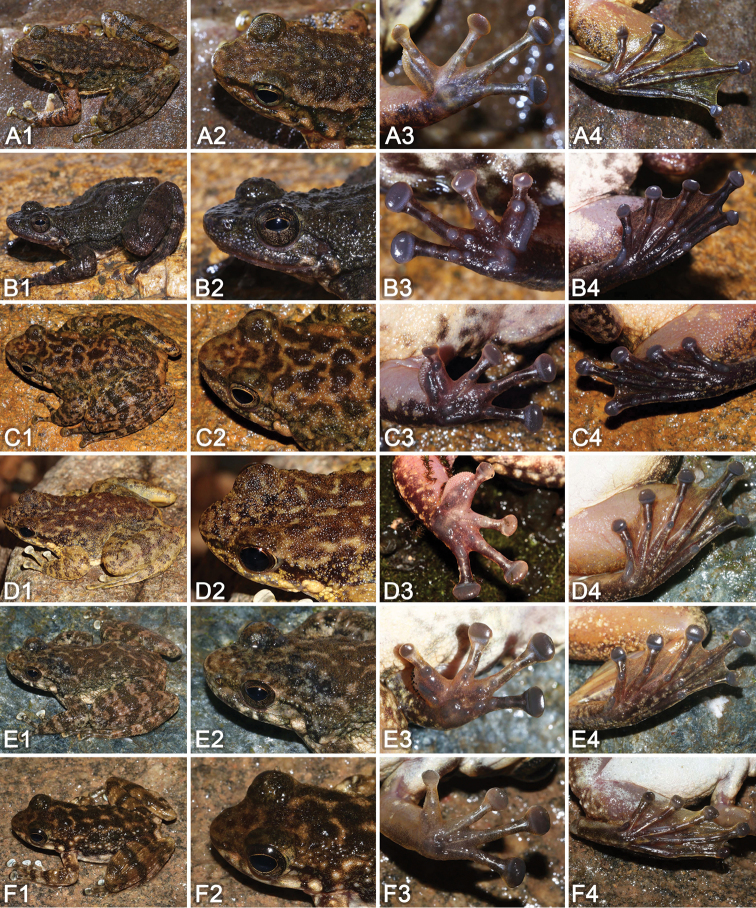
Comparisons of morphological characteristics among species in the *Amolopsricketti* group. **A***A.sinensis* sp. n. **B***A.yatseni* sp. n. **C***A.albispinus***D***A.ricketti***E***A.wuyiensis***F***A.yunkaiensis***1** dorsolateral view **2** close-up of the head **3** ventral view of the hand **4** ventral view of the foot.

The character of longitudinal glandular folds on skin of shoulders makes *Amolopssinensis* sp. n. unique when compared with all known congeners within the genus. The new species is further compared with the four recognized species of the *A.ricketti* species group below (Fig. 5).

*Amolopssinensis* sp. n. was previously reported as *A.ricketti*, but significantly differs from the topotype of *A.ricketti* by the presence of longitudinal glandular folds on the skin of the shoulders (vs. absent), large raised warts on the dorsal surface of body (vs. relatively smooth), the presence of white conical spines on skin of temporal region and loreal region in breeding males (vs. absent), and nuptial pad and nuptial spines beige (vs. white).

*Amolopssinensis* sp. n. is phylogenetically close to *A.albispinus*, but can be distinguished from the later by the presence of longitudinal glandular folds on the skin of the shoulders (vs. absent), the presence of supernumerary tubercles below the base of fingers III and IV (vs. absent), outer metacarpal tubercle slightly separated (vs. completely divided into two tubercles), pineal body distinct (vs. indistinct), ventral surface smooth (vs. presence of tiny, transparent and dispersive conical spines on surface of chest in males), and nuptial spines beige (vs. white).

*Amolopssinensis* sp. n. can be easily distinguished from *A.wuyiensis* by the presence of longitudinal glandular folds on skin of shoulders (vs. absent), vomerine teeth present (vs. absent), lacking vocal sacs (vs. present), and nuptial spines beige (vs. black).

*Amolopssinensis* sp. n. further differs from *A.yunkaiensis* by the presence of longitudinal glandular folds on the skin of the shoulders (vs. absent), larger body size, SVL 40.2–46.5 mm in adult males and 47.7–52.7 mm in adult females (vs. SVL 31.8–34.1 mm in males and 35.2–39.0 mm in females), vomerine teeth present (vs. absent), lacking vocal sacs (vs. present), and ventral surface smooth (vs. presence of tiny transparent spines on surface of chest).

##### Etymology.

The specific name “*sinensis*” refers to “Chinese”, for this new species takes a wide distribution in southern China. We suggest its English common name “Chinese Torrent Frog” and Chinese name “Zhong Hua Tuan Wa (中华湍蛙)”.

##### Distribution and habits.

Currently, the Chinese Torrent Frog is recognized from the Shimentai Nature Reserve and Mt. Nankun in Guangdong, Mt. Dupangling in Guangxi, and Mt. Yangming and Mt. Hengshan in Hunan, which indicates the potential distribution area of *Amolopssinensis* sp. n. is from central Guangdong, to northeastern Guangxi and southwestern Hunan.

*Amolopssinensis* sp. n. inhabits rocky, fast-flowing streams (ca 500–1300 m a.s.l.) surrounded by moist subtropical secondary evergreen broadleaved forests. All individuals were observed from April to August. Males bear nuptial spines from April to July; females bear mature light yellow oocytes from April to August. Nevertheless, much of the ecology and behavior of this species remains unknown.

#### Amolops yatseni

Taxon classificationAnimaliaAnuraRanidae

Lyu, Wang & Wang sp. n.

http://zoobank.org/44B205CF-7C89-40BC-9E20-E9FEED5937C8

##### Chresonymy.

*Amolopsricketti* (Boulenger, 1899): Fei et al. 2009 (Xinyi, Guangdong); Li et al. 2011 (Guangdong); Fei et al. 2012 (Xinyi, Guangdong).

##### Holotype.

**Figure 6.**
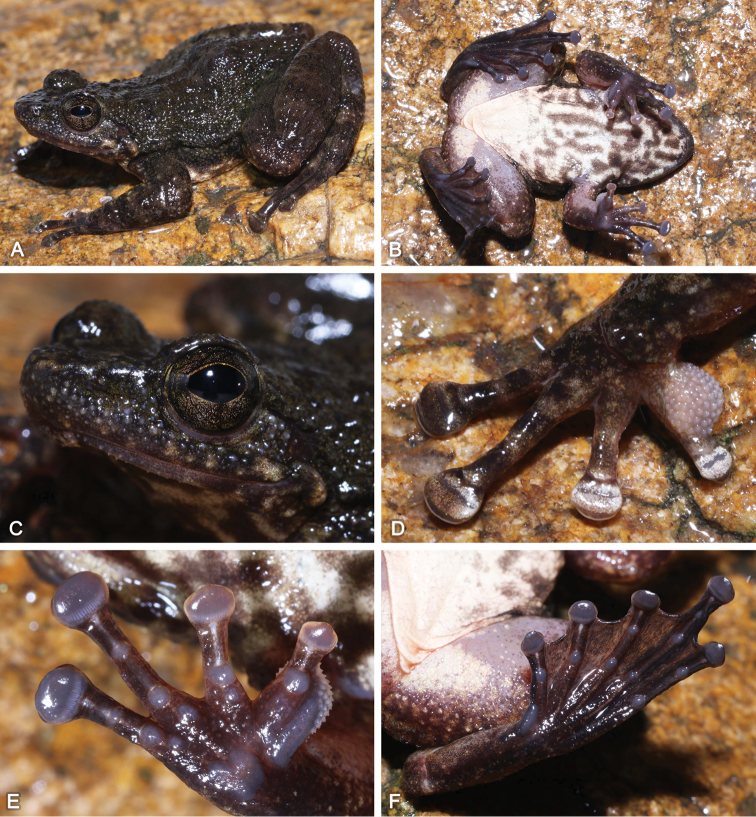
Morphological features of the adult male holotype SYS a006807 of *Amolopsyatseni* sp. n. in life. **A** Dorsolateral view **B** Ventral view **C** Dense white conical spines on skin of temporal region, loreal region, snout, lips and chin **D** Nuptial pad and nuptial spines **E** Right hand **F** Left foot.

SYS a006807 (Fig. 6), adult male, collected by JW and HHC on 27 March 2018 from Mt. Wugui (22.45N, 113.49E; ca 260 m a.s.l.), Zhongshan City, Guangdong.

##### Paratypes.

Sixteen adult specimens. Male SYS a006806, and female SYS a006811/CIB 110005 and SYS a006808–6810, collected at the same time from the same stream as holotype. Male SYS a003634 and 3638, and female SYS a003633, collected by ZTL and JW on 22 April 2015 from Shangchuan Island, Guangdong. Female SYS a006819, collected by JW and HHC on 28 March 2018 from Mt. Gudou, Guangdong. Female SYS a003978 and 3981, collected by ZTL and Chao Huang on 14 May 2015 from Ehuangzhang Nature Reserve, Guangdong. Male SYS a004643 and 4676, and female SYS a004640, 4642 and 4994, collected by ZTL and JW on 14–15 April 2016 from Yunkaishan Nature Reserve, Guangdong.

##### Other examined material.

Juvenile SYS a006857, collected by Yuan-Peng Cen on 26 March 2018 from Daliao, Zhongshan City, Guangdong.

##### Diagnosis.

The new species was assigned to genus *Amolops* and further to the *A.ricketti* group morphologically based on the absence of dorsolateral folds, the presence of a circummarginal groove on the disk of the first finger, the absence of tarsal glands, and the presence of nuptial pads with conical nuptial spines on the first finger in males.

*Amolopsyatseni* sp. n. is distinguished from its congeners by a combination of the following morphological characteristics: (1) body stout and robust, SVL 39.3–44.7 (42.5±2.1, n=6) mm in adult males, 42.1–48.9 (46.4±2.0, n=11) mm in adult females; (2) dorsal body olive-brown or light brown, with irregular light strip-shaped patches or not; (3) ventral surface creamy white, with nebulous dark gray patches or not; (4) dorsal skin of body very rough, granular and scattered with tubercles and raised large warts, lacking warts on central back of trunk in females; (5) dense tiny round translucent, or white, spines present on dorsal skin of body, dorsal and dorsolateral skin of limbs in males, denser in females; (6) vomerine teeth strong, tongue cordiform, deeply notched posteriorly; (7) absence of the dorsolateral folds; (8) supernumerary tubercles below the base of fingers II, III and IV distinct and prominent; (9) heels just meeting; (10) absence of outer metatarsal tubercles and tarsal glands; (11) absence of vocal sacs; (12) nuptial pad on the first finger prominent with developed white conical spines in breeding males, tip of nuptial spines brown; and (13) dense white conical spines present on the skin of the temporal region (including the tympanum in several individuals), loreal region, snout, lips and chin in males during breeding season, and such spines less developed and rounded only on skin of temporal region except the tympanum and lower lips in females.

##### Description of holotype.

Body stout, SVL 41.0 mm. Head width slightly smaller than head length (HDW/HDL = 1.05); snout short (SNT/HDL = 0.38) and rounded in profile, projecting beyond lower jaw; nostril closer to tip of snout than eye; loreal region concave; top of head flat; eye large (ED/HDL = 0.31) and convex; canthus rostralis distinct; pineal body distinct; tympanum small, edge faintly distinct, upper margin of tympanum in contact with supratympanic fold; tympanum-eye distance larger than tympanum, TED/TD 1.08; supratympanic fold distinct, start from back of eye and extending to shoulder; choanae moderate; vomerine teeth present; tongue cordiform, deeply notched posteriorly.

Forelimbs moderately robust; hands moderately long (HND/SVL = 0.31); relative finger lengths I < II < IV < III; finger tips dilated to wide oval disks with circummarginal grooves, relative width of finger disks I < II < III = IV; subarticular tubercles prominent, rounded; supernumerary tubercles below the base of fingers II, III and IV distinct and prominent, below base of fingers I absent; inner metacarpal tubercle elongated and prominent, outer metacarpal tubercle prominent and slightly separated; absence of webbing and presence of weak lateral fringes on fingers.

Hindlimbs long and robust (TIB/SVL = 0.55) ; tibio-tarsal articulation reaching tip of snout when hindlimb stretched alongside of body; relative toe lengths I < II < III = V < IV; tips of all toes expanded to well-developed oval discs with circummarginal grooves; subarticular tubercles oval and distinct; inner metatarsal tubercle prominent, elongated; outer metatarsal tubercles absent; toes fully webbed; lateral fringes of toes I and V developed; tarsal glands absent; heels just meeting when hindlimbs flexed at right angles to axis of body.

Dorsal skin of body very rough, granular and scattered with raised large warts; dense rounded spines present on dorsal body, dorsal limbs, and many well developed and denser ones on sacral region; dense conical spines present on skin of temporal region except tympanum, loreal region, snout, lips and chin, conical spines on skin of lower lips much smaller; flanks very rough and granular with raised warts; dorsal limbs rough with numerous tubercles; several longitudinal dermal ridges on dorsal surfaces of thigh, tibia and tarsus; dorsolateral fold absent; posterior part of upper lip swollen; rictal gland prominent and ellipsoidal, posterior to corner of mouth.

Ventral surface slightly wrinkled with round spines on chest; ventral surface of hand and foot granular; large warts surrounding the vent.

##### Measurements of holotype

**(in mm).**SVL 41.0; HDL 15.3, HDW 16.1; SNT 5.8; IND 5.7; IOD 4.0; ED 4.8; TD 1.7; TED 1.8; HND 12.5; RAD 9.0; FTL 31.2; TIB 22.5; F3W 2.7; T4W 2.1.

##### Color of holotype in life.

Dorsal body dark green; faint dark transverse bars on dorsal surface of limbs; dorsal discs of digits yellowish brown; posterior edge of upper lip and rictal gland light maize-yellow; all round spines and conical spines on skin grayish white; throat, chest, and belly creamy white; several dark gray nebulous mottling on surface of throat, chest and anterior part of abdomen; ventral surfaces of limbs gray pink grounding; creamy white blotches on ventral thighs; rear of thighs mottled with dark brown; ventral hands and feet dark grey; yellowish white tubercles and olive-brown warts around cloaca.

##### Color of holotype in preservative.

Dorsal surface dark brown, irregular light strip-shaped patches present, transverse bars indistinct; ventral surface grayish white, mottling on surface of throat, chest and anterior part of abdomen become more distinct; ventral surface of limbs light brown.

##### Variations.

**Figure 7.**
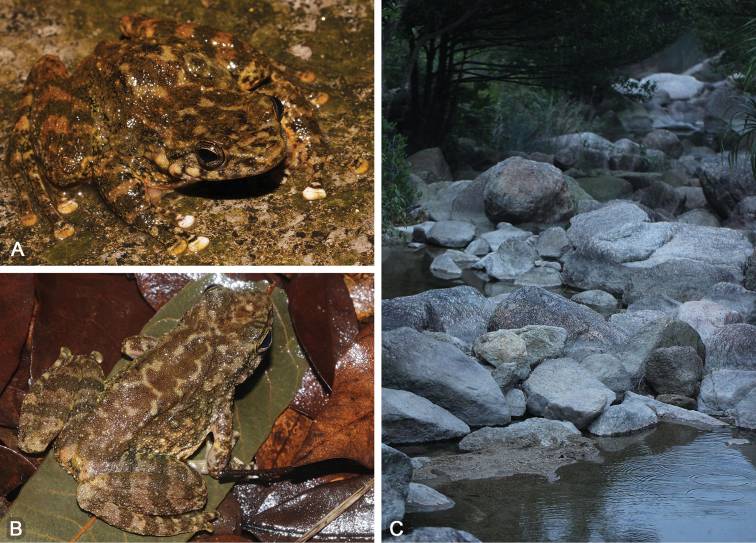
**A** Male paratype SYS a004643 of *Amolopsyatseni* sp. n. in life **B** Female paratype SYS a003981 in life, showing denser and more distinct rounded spines on dorsal skin **C** Habits on Shangchuan Island.

Measurements of type series specimens are given in Table 3. All specimens are very similar in morphology except that: dorsal skin without any patterns in the specimens from Zhongshan City (vs. dorsal skin with irregular light strip-shaped patches in the remaining specimens); skin of tympanum with white conical spines in SYS a004643 (Fig. 7A) and 4676; tibia-tarsal articulation reaching anterior corner of eye in SYS a003633, 3634, 3678, 3680, 4994, 6806 and 6809).

##### Sexual dimorphism.

*Amolopsyatseni* sp. n. possesses significantly-distinct sexual dimorphism: (1) larger body size in females with SVL 42.1–48.9 mm (vs. SVL 39.3–44.7 in males); (2) white nuptial spines with brown tips on white nuptial pads in breeding males; (3) rounded spines on dorsal skin denser and more distinct in females (Fig. 7B); (4) skin of central back bearing raised large warts in males (vs. such warts absent in females); (5) dense white conical spines on skin of temporal region (including the tympanum in several individuals), loreal region, snout, lips and chin in males during breeding season (vs. spines underdeveloped and rounded only on skin of temporal region and lower lips in females); and (6) females bearing light yellow oocytes.

##### Comparisons.

The dense tiny round translucent, or white, spines on dorsal skin of body, dorsal and dorsolateral skin of the limbs makes *Amolopsyatseni* sp. n. unique when compared with all known congeners within the genus. *Amolopsyatseni* sp. n. is further compared with *Amolopssinensis* sp. n. and other four recognized species within the *A.ricketti* species group below (Fig. 5).

*Amolopsyatseni* sp. n. is a sister taxon to *A.sinensis* sp. n. in our phylogenetic trees (Fig. 2), and differs from the later by a significant genetic divergence of 3.5–4.2%. Morphologically, *A.yatseni* sp. n. differs from *A.sinensis* sp. n. by the presence of dense tiny round translucent, or white, spines on the dorsal skin of the body, dorsal and dorsolateral skin of limbs (vs. absent), the presence of rounded spines on the skin of the temporal region and lower-lips in females (vs. absent), the absence of longitudinal glandular folds on the skin of the shoulders (vs. present), supernumerary tubercles below the base of fingers II, III and IV distinct and prominent (vs. indistinct below the base of fingers III and IV, absent below the base of finger II), and heels just meeting (vs. overlapping).

*Amolopsyatseni* sp. n. was previously reported as *A.ricketti*, but significantly differs from the topotype *A.ricketti* by the presence of dense tiny round translucent or white spines on the dorsal skin of the body, dorsal and dorsolateral skin of the limbs (vs. absent), large raised warts on dorsal surface of body (vs. relatively smooth), supernumerary tubercles below the base of fingers II, III and IV distinct and prominent (vs. indistinct below the base of fingers III and IV, absent below the base of finger II), and the presence of white conical spines on skin of temporal region and loreal region in breeding males (vs. absent).

*Amolopsyatseni* sp. n. differs from *A.albispinus* by the presence of dense tiny round translucent, or white, spines on the dorsal skin of the body, dorsal and dorsolateral skin of the limbs (vs. absent), pineal body distinct (vs. indistinct), the presence of conical spines on skin of the tympanum (vs. absent), the presence of rounded spines on skin of temporal region and lower-lips in females (vs. absent), the presence of supernumerary tubercles below the base of fingers II, III and IV (vs. absent), and ventral surface smooth (vs. presence of tiny, transparent and dispersive conical spines on surface of chest in males).

*Amolopsyatseni* sp. n. can be easily distinguished from *A.wuyiensis* by the presence of dense tiny round translucent or white spines on dorsal skin of body, dorsal and dorsolateral skin of limbs (vs. absent), vomerine teeth present (vs. absent), lacking vocal sacs (vs. present), and conical nuptial spines white (vs. black).

*Amolopsyatseni* sp. n. further differs from *A.yunkaiensis* by the presence of dense tiny round translucent or white spines on dorsal skin of body, dorsal and dorsolateral skin of limbs (vs. absent), a significantly larger body size, SVL 39.3–44.7 mm in adult males and 42.1–48.9 mm in adult females (vs. SVL 31.8–34.1 mm in males and 35.2–39.0 mm in females), vomerine teeth present (vs. absent), lacking vocal sacs (vs. present), and ventral surface smooth (vs. presence of tiny transparent spines on surface of chest).

##### Etymology.

The specific name “*yatseni*” refers to the founder of Sun Yat-sen University, Dr. Sun Yat-sen, who was born in Cuiheng Village, Zhongshan City, about five kilometers from the type locality, Mt. Wugui. We suggest its English common name “Yat-sen’s Torrent Frog” and Chinese name “Yi Xian Tuan Wa (逸仙湍蛙)”.

##### Distribution and habits.

Currently, the Yat-sen’s Torrent Frog is known from the Zhongshan City, as well as from Mt. Gudou, Shangchuan Island, Ehuangzhang Nature Reserve, and Yunkaishan Nature Reserve. All these localities are situated in the coastal hills of west Guangdong, indicating the potential distribution area of *Amolopsyatseni* sp. n. is from the west border of Pearl River Delta to the Yunkai Mountains. However, the five known localities of the new species are being threatened by hydropower station construction and tourism development respectively, and surveys are needed in western Guangdong to investigate the accurate population status and the distribution of this species.

*Amolopsyatseni* sp. n. inhabits rocky, fast-flowing streams (ca 250–1000 m a.s.l.) surrounded by moist subtropical secondary evergreen broadleaved forests (Fig. 7C). All individuals were observed from March to August when males bear nuptial spines and females bear mature oocytes. Nevertheless, much of the ecology and behavior of this species remains unknown.

## Discussion

The species *Amolopsricketti* was originally described based on two specimens from Mt. Wuyi, Fujian (Boulenger 1899), and was recorded subsequently over wide area from southern China to northern and central Indochina (Bourret 1942; Liu 1950; Fei et al. 2012; Frost 2018). In this work, we have found that the recorded population of *A.ricketti* from central Guangdong, northeastern Guangxi and southwestern Hunan (now recognized as *A.sinensis* sp. n.) and from coastal hills of west Guangdong (now recognized as *A.yatseni* sp. n.), are markedly different from the topotype of *A.ricketti* from Fujian, both morphologically and genetically. This indicates that the current records of *A.ricketti* might be a species complex (designated here as *A.ricketti* sensu lato) composed of multiple species. Further surveys and studies are required to clarify the concept of *A.ricketti*, especially for the reported populations from southwestern China and Indochina and to determine the accurate distribution of *A.sinensis* sp. n. and *A.yatseni* sp. n.

Southwestern China has been considered as hotspot area with highest species diversity over time, while southeastern China, which suffers from more human activities, is considered as much less diverse, which may reflect the lack of biodiversity surveys over time. Recently, a number of new amphibian species were described from southeastern China (Lyu et al. 2017; Wang et al. 2017; Yuan et al. 2017; Zeng et al. 2017; Lyu et al. 2018; Wang et al. 2018a; Wang et al. 2018b; this study), to be the greatest number of new amphibian species in China in recent times. These discoveries indicate that the species diversity in southeastern China is highly underestimated. Comprehensive and careful surveys are urgently demanded to investigate the biodiversity status in this area, especially for herpetological species which are sensitive to rapid environmental changes.

## Supplementary Material

XML Treatment for Amolops sinensis

XML Treatment for Amolops yatseni

## Appendix 1

**Specimens examined**

*Amolopsalbispinus* (n=10): China: Guangdong Province: Shenzhen City: Mt. Wutong (type locality): SYS a003270–3271, 3452–3454, 4511, 5643; Mt. Paiya: SYS a002436, 6898–6899.

*Amolopsricketti* (n=16): China: Fujian Province: Wuyishan City: Mt. Wuyi (type locality): SYS a004141–4143, 5922–5923; Taining County: Mt. Emeifeng: SYS a002492; Shanghang County: Gutian Town: SYS a003342, 4106; Jiangxi Province: Yanshan County: Wuyishan Nature Reserve: SYS a001605, 1342–1343; Guixi City: Yangjifeng Nature Reserve: SYS a000214, 0240, 0314, 0354–0355.

*Amolopswuyiensis* (n=20): China: Fujian Province: Wuyishan City: Mt. Wuyi (type locality): SYS a001716–1717, 4139–4140, 5936–5938; Shaowu City: Longhu Forest Station: SYS a004129, 4131; Ninghua County: Mt. Yashu: 5897–5900; Jiangxi Province: Yanshan County: Wuyishan Nature Reserve: SYS a001606; Guixi City: Yangjifeng Nature Reserve: SYS a000324, 0358–0360; Guangfeng County: Tongboshan Nature Reserve: SYS a001668; Zhejiang Province: Jingning County: Makeng Village: SYS a002723.

*Amolopsyunkaiensis* (n=21): China: Guangdong Province: Yangchun City: Ehuangzhang Nature Reserve (type locality): SYS a000773–0774, 3979, 3982, 4696–4701, 4703–4707; Xinyi City: Yunkaishan Nature Reserve: SYS a004674, 4681–4684, 4992.
